# Successful management with Paul^®^ Glaucoma Drainage Implant after complicated bleb needling with uveal prolapse into the bleb ten years after trabeculectomy

**DOI:** 10.1186/s12886-025-03906-2

**Published:** 2025-02-24

**Authors:** Ermioni Panidou-Marschelke, Ekaterina Sokolenko, Carsten Framme, Maximilian Binter

**Affiliations:** Department of Ophthalmology, University Eye Hospital, Hannover Medical School, Hannover, Germany

**Keywords:** Uveal prolapse, Bleb needling, Trabeculectomy, Paul^®^ Glaucoma drainage Implant

## Abstract

**Background:**

Fibrosis is the primary cause of failure following glaucoma surgery. Wound healing modulation with 5-fluorouracil and mitomycin-C is routinely employed to reduce ocular fibrosis and improve surgical success rates; however, it also increases the risk of postoperative complications.

**Case presentation:**

A 59-year-old patient with a family history of glaucoma presented a decade after bilateral trabeculectomy with an intraocular pressure (IOP) of 30 mmHg in the right eye and 42 mmHg in the left eye. Both eyes underwent multiple cyclophotocoagulations in the past and showed ocular surface inflammation due to eyedrop intolerance as well as scarred blebs without scleral thinning. Simultaneous bilateral bleb needling reduced IOP to 7 mmHg on the right eye and 12 mmHg on the left eye. The postoperative course of the right eye was favorable with a stable IOP at the low teens. However, IOP of the left eye rose to 34 mmHg within 3 days, accompanied by a uveal prolapse into the bleb. A subsequent vitrectomy with Tutopatch^®^ and anterior chamber washout was performed after 10 days, followed by implantation of the novel Paul^®^ Glaucoma Drainage Implant after sufficient scleral healing. This resulted in a postoperative IOP of 8 mmHg. After 12 months, no eyedrops were required, there were no signs of ocular surface inflammation, and the IOP was stable at 13 mmHg in the right eye and 12 mmHg in the left eye.

**Conclusion:**

This case highlights a rare occasion of scleral thinning leading to perforation with uveal prolapse after needling, 10 years post-trabeculectomy. Likely causes include the use of antimetabolites, cyclodestructive procedures, and chronic conjunctival inflammation from eyedrops. Although needling is typically low-risk, it can lead to complications similar to trabeculectomy. Preoperative screening for scleral thinning using slit lamp and anterior segment OCT is recommended for high-risk patients. The presented two-stage treatment strategy proved successful in managing this complex case.

## Background

Glaucoma is the leading cause of irreversible blindness worldwide and its incidence will grow significantly in the future due to demographic changes related to prolonged life expectancy [1, 2]. When medicinal or laser treatment fails to prevent further ganglion cell loss in the glaucomatous eye, surgery should be considered [3]. The most commonly employed surgical method for treating glaucoma is trabeculectomy, which creates a controlled opening between the anterior chamber of the eye and the space beneath the conjunctiva [3]. The main reason for failure after trabeculectomy, and every other filtrating surgery, remains fibrosis [4, 5]. Wound healing modulation with 5-fluorouracil (5-FU) and mitomycin-C (MMC) is routinely used to attenuate ocular fibrosis thereby improving surgical success rates, but also leads to a rise in postoperative complications [4, 5].

Blebs in eyes with late trabeculectomy failure could be rescued by bleb needle revision with MMC (or 5-FU) which can be effective when performed even up to 30 years after the initial trabeculectomy [6]. Bleb needling is considered a relatively safe procedure with a low incidence of significant complications and rapid post-operative recovery [7].

Kapasi et al. reported a 30% absolute success rate in reducing intraocular pressure (IOP) and a 35% qualified success rate in reducing IOP two years after needling [8]. Lin et al. reported a 36% success rate in achieving a significant reduction in IOP over a three-year period [7]. Complications such as hypotony with choroidal detachments or hyphema were transient and resolved spontaneously during the early postoperative period. However, late hypotony maculopathy was observed in 2% of patients three years after bleb needling [7].

For patients with refractory and complex glaucoma, where other treatments may not be effective, the glaucoma drainage devices are of great importance.

The Paul^®^ Glaucoma Drainage Implant (PGI) is a novel non-valved implant composed of medical-grade silicone. It features a flexible large plate, a small outer and inner tube diameter, therefore exhibiting lower redundant flow capacity than other valveless drainage implants and theoretically minimizing conjunctival erosion [9]. PGI offers a less invasive approach and achieves a more controlled reduction of IOP, making it a better option for patients at higher risk of complications, such as hypotony or corneal decompensation [10, 11].

## Case presentation

A male 59-year-old patient presented to our clinic with bilateral scarred drainage blebs. He has been managing glaucoma for the past 23 years, with a maximum IOP reaching 35 mmHg. Ten years ago, he underwent trabeculectomy in both eyes. In recent months, he has undergone selective laser trabeculoplasty and multiple sessions of micropulse cyclophotocoagulation in both eyes. His conservative treatment regimen includes prostaglandin analogs, carbonic anhydrase inhibitors, β-blockers, and α-agonists for both eyes.

## Investigations

The patient’s best-corrected visual acuity (BCVA) was 20/25 in both eyes, and the IOP measured 35 mmHg in the right eye and 34 mmHg in the left eye. Slit lamp examination of both eyes revealed blepharitis, red conjunctivae with no hint of scleral thinning, limbal injection, scarred blebs at 12 o’clock, open iridotomies, and pseudophakic status. Optic disc examination showed a cup-to-disc ratio of 0.7 in the right eye and 1.0 in the left eye. Optical coherence tomography (OCT) of the discs also indicated a reduction in the peripapillary retinal nerve fiber layer (RNFL). Visual field testing revealed glaucomatous damage the right eye with a mean deviation (MD) of 2.3 dB and severe glaucoma in the left eye with an MD of 14.8 dB.

## Treatment

Needling of the blebs was performed in both eyes using a 25-gauge needle, introduced into the subconjunctival space of the previous filtering bleb, away from the trabeculectomy site. Gentle side-to-side motions were executed to incise and lyse scar tissue. Following the procedure, a subconjunctival injection of 0,1 ml 5-FU (5 mg/ml) was administered. Compression of the elevated bleb away from the cornea temporarily collapsed the bleb, but its reformation confirmed effective aqueous filtration into the subconjunctival space.

**Fig. 1:**
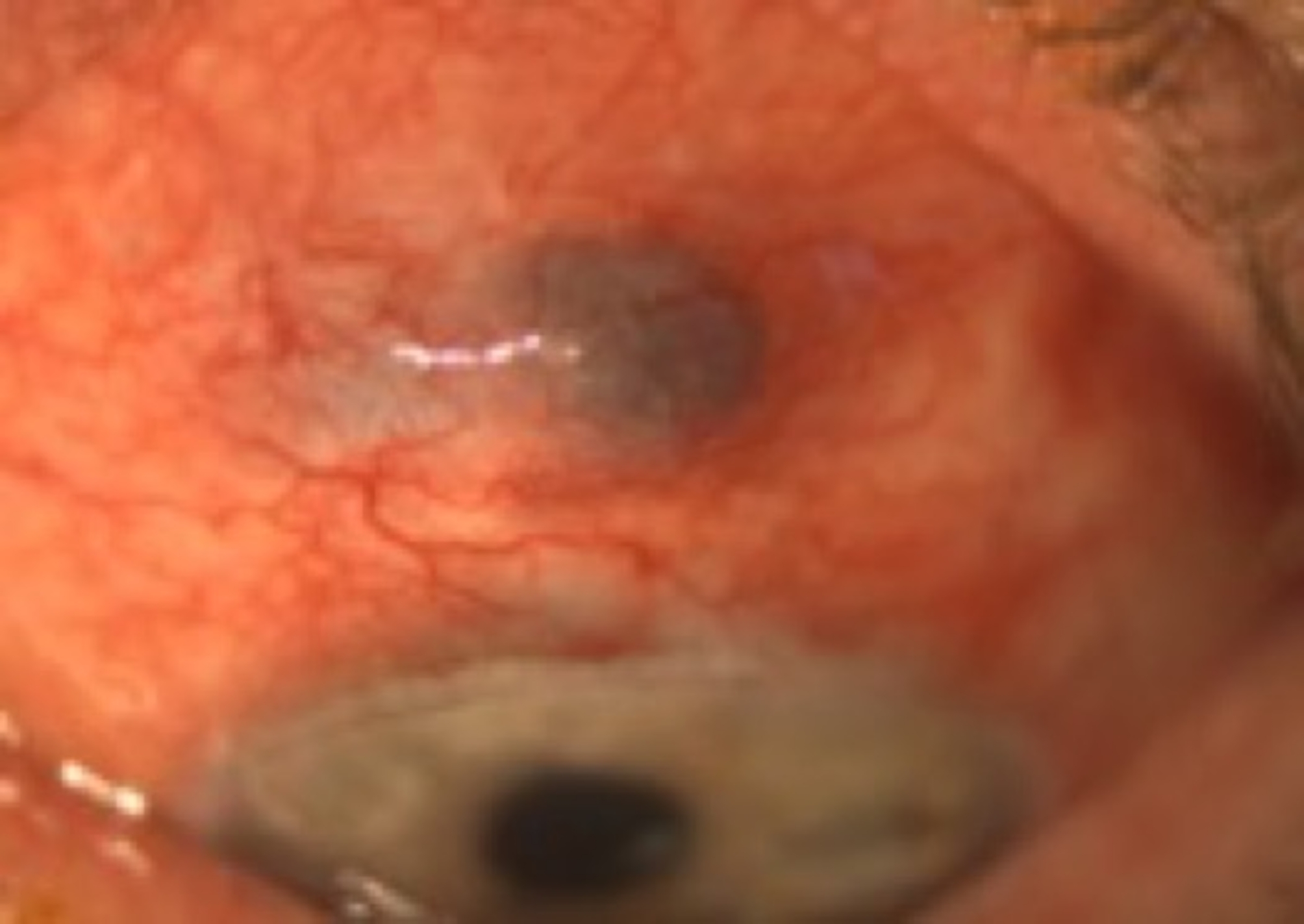
Left eye, 3 days post-needling: A prolapse of dark tissue is visible, indicating choroidal prolapse into the bleb due to scleral thinning

The IOP reached 7 mmHg in the right eye and 12 mmHg in the left eye on the first day postoperatively. Slit lamp examination showed a prominent bleb on the right eye. The left eye also demonstrated a prominent bleb but also a blood clot under the conjunctiva, and erythrocytes in the anterior chamber. Subconjunctival 5-FU injections (5 mg/ml) were administered on both eyes. The clinical course of the right eye was favorable, with IOP remaining around 10 mmHg and a BCVA of 20/32. BCVA in the left eye decreased on the 3rd day to 20/500, while the IOP increased to 34 mmHg. Slit lamp examination revealed a deep anterior chamber with no signs of inflammation, but a prolapse of dark tissue representing the choroid into the bleb (Fig. 1).

**Fig. 2:**
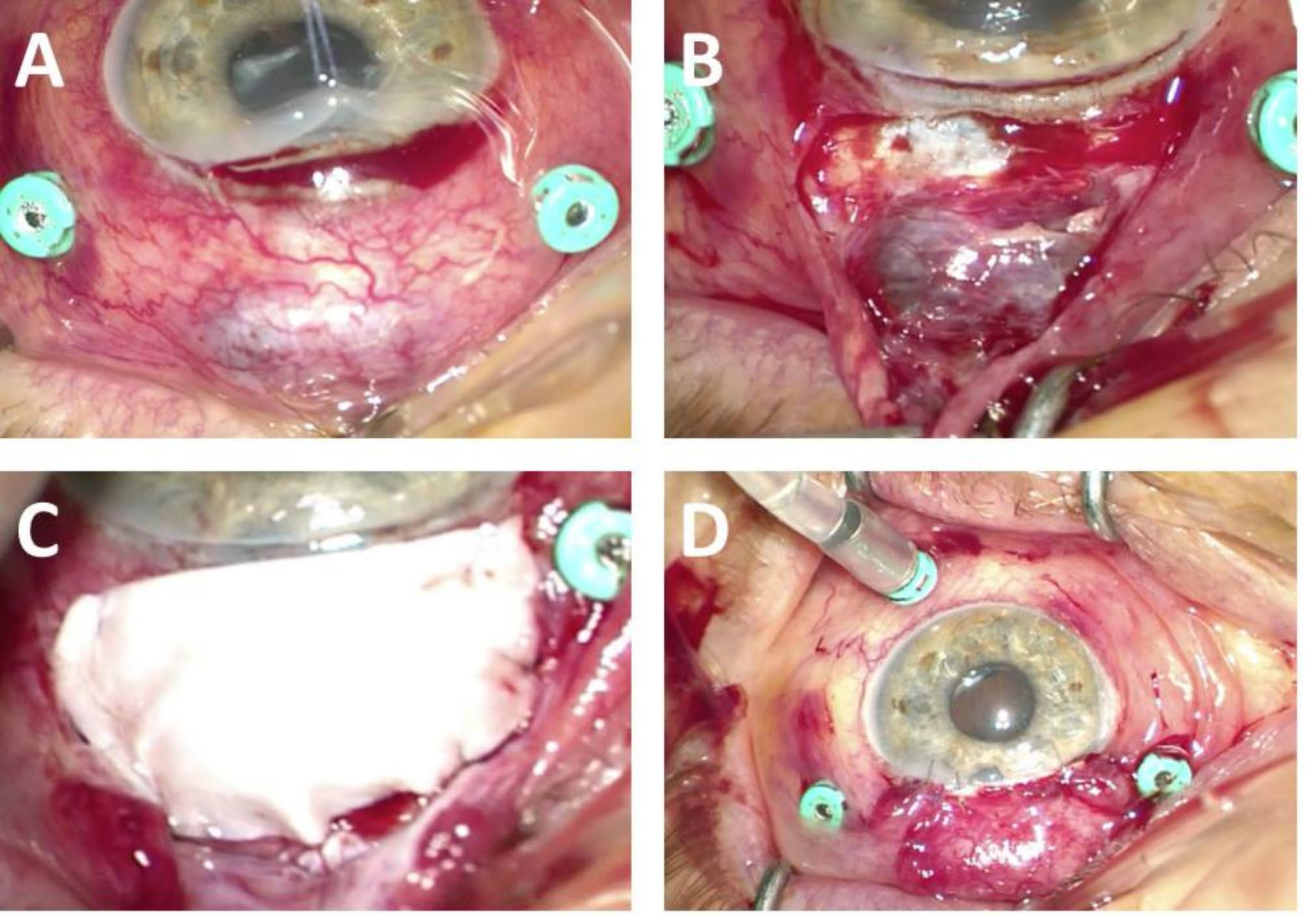
Left eye during emergency surgery: (**a**) corneal melting became apparent after the traction suture; (**b**) significant scleral thinning with uveal prolapse extending into the bleb area; (**c**) defect coverage using Tutopatch^®^ graft; (**d**) the eye at the end of the surgery with closed conjunctiva and corneoscleral sutures

An emergency exploratory operation with vitrectomy revealed a large area of scleral thinning 4 mm from the limbus with choroidal prolapse, and a melted cornea near the bleb with lamellar separation of the peripheral cornea between 11:30 and 12:30. Blood clots were observed in the vitreous cavity, but no suprachoroidal bleeding or retinal detachment was noted. The defect was managed with a Tutopatch^®^ graft and corneoscleral suturing (Fig. 2).

10 days postoperatively the IOP began to rise again, reaching values in the mid-20s range. In order to facilitate the integration of the Tutopatch^®^ graft, the patient was temporarily prescribed oral carbonic anhydrase inhibitors (CAIs) twice daily. Four weeks post-operation, the sclera exhibited satisfactory healing with no signs of scleral thinning. However, despite the oral CAIs, the IOP was 42 mmHg, prompting the decision to proceed with implantation of a glaucoma drainage implant.

The Paul^®^ Glaucoma Drainage Implant was placed in the inferior-temporal quadrant. After adequate dissection of the conjunctiva and Tenon’s capsule, the PGI endplate was placed under the rectus muscles and sutured to the sclera with 9 − 0 Ethilon^®^ suture 11 mm posterior to the limbus. Part of a 6 − 0 Prolene^®^ suture was placed in the PGI tube. The tube was trimmed to a few millimeters within the anterior chamber, with the bevel oriented upward. A 26-gauge needle was used to create the anterior chamber entry through the limbus, parallel to the iris plane. The tube was inserted into the anterior chamber through the needle pathway and positioned just above the iris and away from the corneal endothelium. The tube was covered along its length with Tutopatch^®^ and secured to the sclera using 8 − 0 Vicryl^®^ sutures, and the conjunctiva was closed with the same material.

10 days after the PGI implantation, the IOP rose back up to 25 mmHg, and slit lamp examination revealed an already cystic and scarred bleb. An open revision of the bleb was performed the following day. In the following, the patient was discharged with an IOP of 11 mmHg and a BCVA of 20/30. The entire anterior segment of the eye appeared normal, with no signs of inflammation.

## Outcome and follow-up

**Fig. 3:**
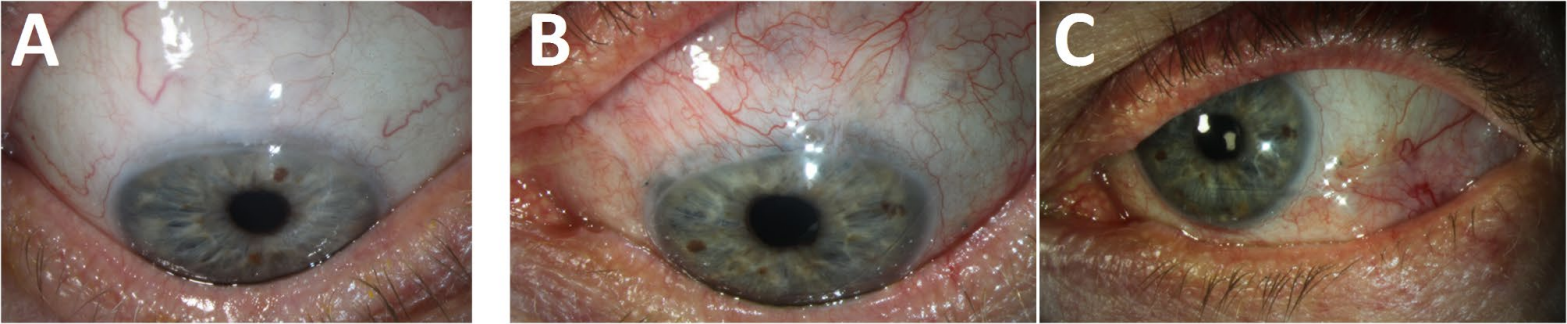
Findings after 6 months: (**a**) Right eye in downgaze with prominent bleb; (**b**) left eye in downgaze with the Tutopatch^®^ well integrated and (**c**) in right gaze with filtering Paul^®^ Glaucoma Drainage Implant

After 12 months BCVA was 20/20 in both eyes and IOP measured 13 mmHg in the right eye and 12 mmHg in the left eye, without any local therapy in both eyes (Fig. 3). In comparison to the initial examination, endothelial cell count remained stable at 1976 cells/mm2 in the right eye but decreased from 1988 to 1560 cells/mm2 in the left eye. Furthermore, there was no reduction in global retinal RNFL thickness in comparison to the initial examination for both eyes. Additionally, the mean deviation in perimetry did not decrease in the left eye; only a slight change from 2.4 dB to 4.8 dB was observed in the right eye.

## Discussion

To our knowledge, there is only one case in the literature showing scleral thinning leading to perforation in the area of the bleb with uveal tissue blockage after needling. However, there are several differences. Firstly, in that case, the blockage was caused by a known mechanism, specifically by iris tissue. Secondly, this occurred with a significant latency of 22 months after needling [12]. These two points limit the comparability of the cases. However, both cases demonstrate that in the event of a perforated scleral thinning after needling, a successful two-stage treatment with an initial scleral patch graft and, after healing, a glaucoma drainage implant is possible [12, 13].

The uveal prolapse in this case was likely caused by the mechanical irritation from needling in a high-risk eye with pronounced, previously undetected scleral thinning. This scleral thinning of the eye was probably due to several risk factors. Firstly, antimetabolites like MMC pose a risk for scleral thinning, which is documented in the literature as a rare but known side effect [4, 5]. Secondly, the patient had already undergone several CPC procedures on the affected eye, and scleral thinning is also recognized as an undesirable consequence of these interventions [14]. Moreover, inflammation of the conjunctiva and the chronic use of local anti-glaucomatous and corticosteroid eye drops could have contributed to the scleral thinning [4, 5].

This serves as a pertinent reminder that while needling is typically considered a procedure with few complications, it still carries similar risks to trabeculectomy and should be treated as such.

It is essential to critically evaluate the necessity of needling, particularly in cases with a significant history of prior extensive MMC usage and post-CPC procedures. It is imperative to adopt a comprehensive approach, considering the eye as a whole and taking into account its complete medical history. Moreover, screening for thinned sclera should be performed prior to needling in high-risk eyes. In addition to a thorough slit-lamp examination, a preoperative OCT of the sclera can also be performed to assess scleral thickness [15].

Additionally, it is encouraging to note that even in long-standing scarring after trabeculectomy, such as in this case with a 10-year-old bleb after trabeculectomy, needling remains a viable and successful therapeutic option, as demonstrated by the right eye’s outcome in this patient. It is noteworthy that this success was achieved despite previous CPC treatments on the eye. This highlights the importance of attempting revision or needling before resorting to CPC. Despite its lower overall success rate for late trabeculectomy failure, needling is still recommended as the initial approach [7, 13].

Paczka et al. have demonstrated the effective reduction of intraocular pressure using the Ahmed Valve in their case [12]. Similarly, the newly introduced Paul^®^ Glaucoma Implant, used in this case, proved effective in managing a complex scenario, further supporting its efficacy in such challenging situations.

The most commonly used and studied glaucoma drainage devices are the Ahmed Glaucoma Valve (AGV) and the Baerveldt Glaucoma Implant (BGI). Studies suggest that the 2-year cumulative failure rates for AGV (48.4%) and BGI (44.2%) are significantly higher than those for PGI, which has a notably lower failure rate of 17.8% [9, 16]. The high failure rates of the AGV and BGI could be explained by the external and internal calibers of the PGI, which are over 30% and 50% smaller than those of the BGI and AGV, respectively, resulting in a flow-restrictive advantage for the PGI [9]. Studies also highlighted the importance of plate dimensions, linking a larger plate surface area and flatter plate profile to a lower risk of encapsulation and therefore higher success rates [9, 17]. The PGI addresses this by having a shorter wingspan but a longer posterior plate extension which increases the effective surface area [9]. These theoretical benefits in the design of the PGI have been used to explain its superior performance relative to the AGV and BGI [9].

The smaller tube caliber is theoretically associated with a reduced risk of tube-endothelium contact and subsequent damage [9]. The AVB study reported central endothelial damage rates of 3% and 14% one year after implantation of AGV and BGI, respectively [16]. Data for the first year following PGI implantation are particularly favorable, with some studies reporting damage rates as low as 0% [18, 19]. However, longer-term studies indicate higher rates, with 5.4% reported after two years in one study and 3.8% after three years in another [11, 20].

Berteloot et al. conducted a retrospective study comparing the efficacy and safety of the BGI and PGI, demonstrating that the PGI provided better IOP control during the early postoperative period [19]. This was also a key factor for selecting a PGI for the patient in the present case report.

This case report has certain limitations, including the short duration of follow-up and its focus on a single patient. Further long-term data with a larger cohort of patients are needed to better evaluate the effectiveness of this two-stage treatment procedure.

## Conclusion

Although needling is typically low-risk, it can lead to complications similar to trabeculectomy. Preoperative screening for scleral thinning using slit lamp and anterior segment OCT is recommended for high-risk patients. The two-stage treatment strategy utilized in the presented case was successful and may be considered in similar situations.

## Data Availability

No datasets were generated or analysed during the current study.
